# Regulation of the *vapBC-1* Toxin-Antitoxin Locus in Nontypeable *Haemophilus influenzae*

**DOI:** 10.1371/journal.pone.0032199

**Published:** 2012-03-13

**Authors:** Susan D. Cline, Sehresh Saleem, Dayle A. Daines

**Affiliations:** Division of Basic Medical Sciences, Mercer University School of Medicine, Macon, Georgia, United States of America; University of Padova, , Italy

## Abstract

Nontypeable *Haemophilus influenzae* (NTHi) are human-adapted commensal bacteria that can cause a number of chronic mucosal infections, including otitis media and bronchitis. One way for these organisms to survive antibiotic therapy and cause recurrent disease is to stop replicating, as most antimicrobials target essential biosynthetic pathways. Toxin-antitoxin (TA) gene pairs have been shown to facilitate entry into a reversible bacteriostatic state. Characteristically, these operons encode a protein toxin and an antitoxin that associate following translation to form a nontoxic complex, which then binds to and regulates the cognate TA promoter. Under stressful conditions, the labile antitoxin is degraded and the complex disintegrates, freeing the stable toxin to facilitate growth arrest. How these events affected the regulation of the TA locus, as well as how the transcription of the operon was subsequently returned to its normal state upon resumption of growth, was not fully understood. Here we show that expression of the NTHi *vapBC-1* TA locus is repressed by a complex of VapB-1 and VapC-1 under conditions favorable for growth, and activated by the global transactivator Factor for Inversion Stimulation (Fis) upon nutrient upshift from stationary phase. Further, we demonstrate for the first time that the VapC-1 toxin alone can bind to its cognate TA locus control region and that the presence of VapB-1 directs the binding of the VapBC-1 complex in the transcriptional regulation of *vapBC-1*.

## Introduction

Unencapsulated (nontypeable) *Haemophilus influenzae* (NTHi) are Gram-negative obligate bacteria of the human upper respiratory tract that have the potential to cause mucosal diseases such as otitis media, bronchitis, and pneumonia [1]. These infections can be recurrent, and NTHi have evolved mechanisms to persist between episodes [2]. One successful survival strategy is the ability to undergo reversible growth arrest, mediated by toxin-antitoxin (TA) gene pairs [3]. TA loci have been found in nearly all bacterial genomes sequenced to date, and many code for a protein toxin and antitoxin that form a nontoxic complex upon translation which autorepresses the cognate promoter. Under stressful environmental conditions such as nutrient limitation, antibiotic therapy, or oxidative stress, the labile antitoxin is degraded and the more stable toxin is freed to facilitate growth arrest. Given that these events would destroy the autoregulatory protein complex, the means by which TA promoter regulation is returned to its normal state upon removal of stress and resumption of growth is not fully understood.

TA gene pairs have been divided into several families, of which the *vapBC* locus is by far the most numerous [4]. This remarkable conservation of *vapBC* is underscored by the fact that many microorganisms contain multiple *vapBC* loci. For example, one study found that 20 *vapBC* TA homologues identified in the *Mycobacterium tuberculosis* genome were active when expressed in *Mycobacterium smegmatis* [5]. NTHi strains maintain two *vapBC* alleles in their chromosomes (designated *vapBC-1* and *vapBC-2*), making this organism an excellent model in which to study the regulation and function of these highly conserved gene pairs.

The VapC toxins are characterized by a 100 amino acid motif known as a PilT N-terminus (PIN) domain that contains highly conserved acidic residues shown to be essential for metal ion coordination in other PIN-domain containing proteins that are ribonucleases [6]. The PIN domains display similarity to the nuclease domains of Taq polymerase, T4 RNase H, and the 5′-3′ flap endonucleases [7]. The VapB antitoxins are thought to have evolved to block the ribonucleic activity of their associated VapC toxins. This is consistent with a study showing that the VapB antitoxins were specific for their cognate toxins in four separate *vapBC* loci in *M. tuberculosis* [5]. However, another study found that certain noncognate *M. tuberculosis* toxin and antitoxin homologues could associate, both *in vivo* and *in vitro* [8], so questions remain about the exact role of each antitoxin. VapB-1 is a member of the VagC superfamily of virulence-associated and related proteins, and contains a SpoVT/AbrB-like domain [9]. AbrB is an ambiactive transcriptional regulator important during the transition from vegetative to the sporulation state in *Bacillus*, and SpoVT is another member of this family with transcriptional regulation activity. Therefore, the VapB-1 antitoxin may serve to control genes involved in stress survival.

We have shown previously that NTHi *vapBC-1* encodes a VapB-1 antitoxin that binds to and interferes with the activity of its cognate ribonuclease VapC-1 toxin. This toxin has a PIN domain and degrades RNA but not single- or double-stranded DNA *in vitro* [10]. However, we have yet to determine how the VapBC-1 complex may control its own expression and modulate the toxic VapC-1 ribonuclease function following stress.

In an earlier study, we also reported that the activity of *vapBC-1* promoter::*lacZ* reporter gene fusions from two NTHi clinical isolates analyzed over the cell cycle in an *Escherichia coli* background displayed an inverse relationship to culture density [10]. This pattern of transcriptional activity is consistent with *vapBC-1* locus regulation by the Factor for Inversion Stimulation (Fis), but Fis expression in NTHi had not been characterized, limiting our interpretation of this data [11]. Fis is a small nucleoid-associated protein that binds to sites throughout bacterial chromosomes and regulates a wide array of genes, both directly by promoter interaction and indirectly by altering DNA structure during various growth stages [12]–[16]. In *Escherichia coli*, the cellular levels of Fis are dramatically increased upon nutrient upshift from stationary phase in batch culture, conditions that would precede TA locus upregulation [11]. A high-affinity binding sequence (5′-GNNYAWWWWWTRNNC-3′, where W is A/T) has been characterized for *E. coli* Fis; however, the Fis binding sequence for *E. coli* and *Salmonella* is rather degenerate, suggesting significant variation in bacterial Fis recognition of DNA [14], [17]–[19]. We have identified a putative Fis binding site within the NTHi *vapBC-1* locus control region that matches 13 of 15 residues of the *E. coli* sequence, suggesting its possible regulation by the NTHi Fis protein.

To gain understanding of the growth phase regulation of the *vapBC-1* operon, we have conducted *in vivo* studies of the *fis* and *vapBC-1* response to nutrient upshift in a NTHi clinical isolate, strain R2866, with the hypothesis that Fis and the VapBC-1 TA complex serve to regulate *vapBC-1* expression. Additionally, the NTHi Fis and VapBC-1 TA proteins were purified and analyzed for their interaction with the *vapBC-1* locus control region. The work presented herein elucidates a novel role for the VapC-1 toxin and supports a new model for *vapBC-1* TA locus autoregulation.

## Materials and Methods

### Bacterial strains and culture conditions

The bacterial strains and plasmids used in these studies are listed in Table 1. *E. coli* strains were grown in LB broth or agar ±30 µg/ml kanamycin or 100 µg/ml ampicillin, as required. NTHi strains were grown in either brain-heart infusion (BHI) broth or agar supplemented with 10 µg/ml heme-histidine and β-NAD (sBHI), or on chocolate agar plates containing 5 kU bacitracin/L. Where required, the following antibiotics were added: 5 µg/ml gentamicin, 30 µg/ml kanamycin, or 2 µg/ml chloramphenicol.

**Table 1:** Bacteria and plasmids used in this study.

Bacteria	Description	Source
***H. influenzae***		
R2866	Blood isolate from an immunocompetent child with meningitis immunized with the Hib vaccine.	A.L. Smith
R2866 Δ*vapBC-1*	The *vapBC-1* TA locus replaced with a kanamycin resistance cassette.	This work
R2866 Δ*fis*	The *fis* gene replaced with a chloramphenicol resistance cassette.	This work
R2866 Δ*fis*Δ*vapBC-1*	*fis* and *vapBC-1* double mutant.	This work
***E. coli***		
BL21	F^−^ *dcm ompT hsdS*(r_B_^−^ m_B_^−^) *gal* [*malB* ^+^]_K-12_(λ^S^)	EMD Bioscience
BL21(DE3)	F^−^ *ompT hsdS* (r_B_^−^m_B_^−^) *gal dcm* λDE3	EMD Bioscience
DD12	MC4100 *recA*::*RP4-2-Tc::Mu* Km^R^	[23]
DH5α	F^−^ *endA1 glnV44 thi-1 recA1 relA1 gyrA96 deoR nupG* Φ80d*lacZ*ΔM15 Δ(*lacZYA-argF*)U169, hsdR17	Lab collection

Plasmids	Description	Source
pBluescript	Cloning vector encoding ampicillin resistance.	Stratagene
pDD514	RSF1010-based *H. influenzae* conjugal expression vector encoding gentamicin resistance.	[23]
pDD686	pET24b with R2866 *vapBC-1*.	[10]
pDD690	pET24b with R2866 *vapB-1*.	[10]
pDD693	pMC1403 with R2866 *vapBC-1* promoter fusion to *lacZ*.	[10]
pDD715	pTrcHisA with R2866 *fis*.	This work
pDD717	pBluescript with the 340 bp R2866 *vapBC-1* locus control and upstream region.	This work
pDD727	pDD514 with R2866 *vapBC-1* promoter fusion to *lacZ*.	This work
pDD731	pGEM5 with R2866 *vapBC-1* deletion marked with a kanamycin cassette.	This work
pDD733	pBluescript with R2866 *fis* deletion marked with a chloramphenicol cassette.	This work
pDD780	pET24b with the *vapC-1* D99N site-directed mutant for protein expression.	This work
pET24b	C-terminal polyhistidine tag expression vector.	EMD Bioscience
pGEM5	Cloning vector encoding ampicillin resistance.	Promega
pMC1403	Promoter::*lacZ* fusion vector.	[22]
pTrcHisA	N-terminal polyhistidine tag expression vector.	Invitrogen

### Cloning of *vapB-1*, *vapBC-1* and *fis*

Each wild-type *vap* gene was cloned into the expression vector pET24b as a C-terminal polyhistidine tag fusion either in single copy (*vapB-1*) or in tandem (*vapBC-1*) as described previously [10]. To determine whether the polyhistidine tag interfered with function, VapB-1 was also cloned with an N-terminal polyhistidine tag in the vector pTrcHisA, which was subsequently removed using the Enterokinase Cleavage Capture Kit (Novagen) according to the manufacturer's instructions. The crystal structure for *E. coli* Fis has been solved [20], and the first 23 amino acids of the homodimer are disordered, whereas the C-terminal domain is considered important for DNA binding. Therefore, we chose an N-terminal polyhistidine tag fusion to express and purify NTHi Fis using the vector pTrcHisA. This tag was subsequently removed using enterokinase cleavage as above. All PCR reactions were performed with Phusion FLASH high-fidelity DNA polymerase (New England Biolabs) using purified genomic DNA from NTHi strain R2866 as the template. Oligonucleotides used in this study were custom synthesized by Eurofins MWG Operon. Each pET24b-based construct was expressed in *E. coli* BL21(DE3). The *fis* gene was amplified from NTHi strain R2866 genomic DNA using high-fidelity PCR with the primers FisXhoIfor and FisKpnIrev (Table 2). The 315 bp product was cut with *XhoI/KpnI* and ligated to *XhoI/KpnI*-cut pTrcHisA, resulting in pDD715, the expression plasmid for Fis with an N-terminal polyhistidine tag. This construct was confirmed by DNA sequencing and expressed in *E. coli* BL21.

**Table 2:** Primers for *fis* and *vapBC-1* cloning.^a^

FisXhoIfor	5′-ATAACTCGAGATGTTAGAACAACAACG-3′
FisKpnIrev	5′-AAACGGTACCTTATCCCATACCG-3′
D99NFor	5′-[Phos]AATCTATGGATCGCTTGTCACG-3′
D99NRev	5′-[Phos]ATTATTTCCGATAGGTCGCC-3′
VapCSacFor	5′-CGAGGAGCTCTATGATTTATATGTTAGACACC-3′
VapCXhoIRev	5′-GAATCTCGAGTTTTGTCCAATCTTGCC-3′
BCFor	5′-GCTTTCTAGACAGGCTAAATATACCG-3′
BCRev	5′- GGTCTCTAGAGGCATTGTGCGCCAC -3′
Fis28Sacfor	5′-AAAAGAGCTCAATGTGCCAGTGACC-3′
Fis28Xbarev	5′-AATATCTAGACCGTTAAGGCATCAGCAGG-3′
Fis28Xbafor	5′-CGTGTCTAGAATATGCTTGGTATCAACCG-3′
Fis28Pstrev	5′-GCACCTGCAGTTTACTGGAAAAGTGC-3′
CmNhefor	5′-GCAGGCTAGCCCGACGCACTTTGC-3′
CmNherev	5′-CTTAGCTAGCTTACGCCCCGCCCTG-3′
BC1403for	5′-ACTAGAATTCATCATTTACTCATTGACTTGC-3′
BC1403rev	5′-GTTAGGATCCTGAAACACTTTAGTAAGC-3′
1403Xbafor	5′-ATTTTCTAGAGTTATTGTCTCATGAGCGG-3′
1403Xbarev	5′-TTCCTCTAGAGAAATACGGGCAGACATGG-3′

a Restriction enzyme sites used for cloning are underlined.

### Site-directed mutagenesis of VapC-1

The aspartate residue at position 99 in VapC-1 was changed to an asparagine by PCR mutagenesis of pDD686 using the following primers: D99NFor and D99NRev (Table 2). The PCR reaction was incubated with *DpnI* to digest the template DNA, and the column-purified product was used to transform competent DH5α cells. The resulting plasmid was sequenced on both strands to ensure the correct D99N mutation, and designated pDD758. To express the VapC-1 D99N mutant alone, pDD758 was amplified with the primers VapCSacFor and VapCXhoIRev and the 660 bp amplicon was cut with *SacI/XhoI* and ligated to *SacI/XhoI*-cut pET24b, creating pDD780 (Table 2). This plasmid was used to express the VapC-1 D99N protein with a C-terminal polyhistidine tag.

### Protein purification

Proteins were purified from induced cultures using the Promega MagneHis protein purification system. Briefly, *E. coli* strains BL21 (for the pTrcHis vector-based constructs) or BL21(DE3) (for the pET24b vector-based constructs) carrying the various fusions were grown to logarithmic phase in LB broth with appropriate antibiotics and induced for 2 or 3 hours with 1 mM IPTG. The cells were pelleted, frozen at −80°C, and subjected to 3 freeze-thaw cycles prior to being processed using the manufacturer's protocol for native purification. Purified proteins were quantitated using the Bradford protein assay (Pierce). Aliquots of purified proteins were frozen at −80°C and thawed when needed. Where required, polyhistidine N-terminal tags were removed by enterokinase cleavage.

### Deletion of *vapBC-1* and *fis*

The *vapBC-1* toxin-antitoxin locus was deleted from strain R2866 by allelic exchange as follows. A 2557 bp area around the *vapBC-1* locus in the R2866 genome was amplified by Phusion high fidelity PCR using the primers BCFor and BCRev (Table 2). The amplicon was cut with *XbaI* and ligated to compatible ends in *SpeI*-cut pGEM5, creating pDD730. The pDD730 plasmid was then cut with *BamHI* and *BglII* and gel purified, which removed 564 of the 619 bp of the *vapBC-1* operon. A 1275 bp Phusion PCR amplicon of the *aphII* gene from pUC4K with *BamHI* ends was ligated in the place of the *vapBC-1* operon, creating pDD731. This plasmid was then amplified with primers that flanked the multiple cloning site, and the amplicon was used to replace the *vapBC-1* operon from strain R2866. PCR of the flanking regions followed by DNA sequencing confirmed the *vapBC-1* deletion.

The *fis* gene was deleted in the following manner. Attempts to clone the region surrounding the NTHi *fis* gene were unsuccessful, so a three-step cloning technique was used. For the first step, a 637 base pair fragment upstream from *fis* which included 40 base pairs into the *fis* coding region was amplified by PCR with the primers Fis28Sacfor and Fis28Xbarev (Table 2). This was cut with *SacI/XbaI* and ligated into the *SacI/XbaI*–digested vector pBluescript SK^+^, creating pDD729. For the second step, pDD729 was cut with *XbaI/PstI* and ligated to a *XbaI/PstI*-cut 317 base pair fragment representing the last 71 base pairs of *fis* plus flanking DNA amplified by PCR using the primers Fis28Xbafor and Fis28Pstrev, creating pDD732. Finally, pDD732 was cut with *XbaI* and ligated to a compatible *NheI*-cut 900 base pair fragment amplified with the primers CmNhefor and CmNherev (Table 2) that contained the chloramphenicol acetyltransferase (*cat*) resistance cassette from pACYC184, resulting in pDD733. This strategy deleted 188 bp of the 299 bp *fis* gene and inserted a *cat* cassette in its place. PCR of the flanking regions followed by DNA sequencing confirmed the *fis* deletion. Use of a high-fidelity PCR amplicon of this region in allelic exchange reactions successfully deleted *fis* from strains R2866 and R2866 *vapBC-1*.

### Quantitative real time-polymerase chain reaction

The expression of *fis* in the wild type NTHi strain R2866 following nutrient upshift was analyzed by qRT-PCR. A 1.5 mL aliquot of a stationary phase culture of strain R2866 in sBHI was diluted into 25 mL of fresh, pre-warmed medium. Culture samples of 1 ml were obtained immediately prior to inoculation (time zero, stationary phase), 10, 30, 60, and 120 minutes following nutrient upshift. Each was immediately added to an equal volume of RNALater (Ambion) and held at 4°C until used (in all cases, less than 2 days). Total RNA from each sample was purified using the Charge Switch Total RNA kit (Invitrogen) according to the manufacturer's instructions, with a modification of changing the DNase I digestion step to 30 min at 37°C. Purified total RNA concentration was then quantified by NanoDrop spectrophotometry (Thermo Scientific), and each RNA sample was used as the template for PCR to ensure that there was no genomic DNA contamination prior to cDNA synthesis. 250 ng of total RNA from each sample was used for cDNA synthesis utilizing the Fermentas Maxima Universal First Strand cDNA Synthesis Kit with the included random hexamer primers. Each cDNA reaction was then diluted 1∶10 and used as template for PCR to ensure that the cDNA had successfully been synthesized. For real-time PCR, 25 µL reactions were prepared with 12.5 µl of 2× Power SYBR Green Master Mix (Applied Biosystems), 400 nM of each gene-specific primer set, and 5 µL of a 1∶10 dilution of cDNA. qPCR was performed using the Applied Biosystems 7300 Real-Time PCR System with the following cycling conditions: 10 min at 95°C, 40 cycles of 95°C for 15 sec and 60°C for 1 min. Relative expression of *fis* was calculated by the comparative C_t_ method (2^–ΔΔCt^) using time zero as the calibrator and the expression of a DNA gyrase subunit gene, *gyrA*, as the endogenous control. The *gyrA* gene was chosen because it had been used previously in qRT-PCR studies for this purpose in NTHi [21]. Primers for *fis* (forward: 5′-AGC AAA CCA TTA CGC GAT TC-3′, reverse: 5′-TTT GCT GCA CGA GTT TGA T-3′) and *gyrA* (forward: 5′-AGG TGT TCG CGG TAT CAA AC-3′, reverse: 5′-ATT GCA CCT TCA CCT TTT TGG-3′) were designed based on the genome sequence of R2866 using the Primer3 software (http://frodo.wi.mit.edu/primer3/). Three biological replicates and three technical replicates were analyzed for each time point.

### Promoter::reporter gene fusions and activity assays

For the *lacZ* fusion, a 340 base pair sequence located upstream of the *vapBC-1* locus in strain R2866 that included coding sequence for the first seven amino acids of *vapB-1* was amplified by PCR with the primers BC1403for and BC1403rev, which included engineered *EcoRI* and *BamHI* sites, respectively (Table 2). This fragment was then ligated in-frame with a promoterless *lacZ* reporter gene in the vector pMC1403 [22], creating pDD693. To express this promoter fusion in NTHi, pDD693 was used as the template for PCR with the primers 1403Xbafor and 1403Xbarev. The amplicon was cut with *Xba*I and ligated to the compatible ends of the *NheI*-cut broad host-range conjugal plasmid pDD514 [23], resulting in pDD727. This plasmid was then conjugated into strain R2866 and the deletion mutants, and promoter activity was measured via β-galactosidase activity assays performed at least three times in triplicate at time zero (stationary phase) and every 30 minutes for the first 2 hours following nutrient upshift. The algorithm for determining β-galactosidase activity is: [OD_420_−(1.75*OD_550_)/t*v*OD_600_]*1000, where t = time of development of the reaction in minutes, v = volume of the sample in milliliters, and OD_600_ is the measure of the culture density [24]. This equation allows normalization of culture densities for comparison purposes.

### DNase I protection analysis

A 153 bp substrate consisting of the *vapB-1* translation initiation region and upstream sequence from the 340 bp of R2866 *vapBC-1* locus control region above was produced by PCR amplification of pDD717 using high-fidelity Phusion FLASH DNA polymerase and the forward EcoRIBCpromfor, 5′- TTA GAA TTC GCT CGA TGA TTG CGG-3′ and reverse BC1403rev (see Table 2) primers at equimolar concentration. To enable the detection of DNase I products arising from the sense strand of the 153 bp *vapBC-1* substrate, the forward primer was 5′-labeled with ^32^P using T4 polynucleotide kinase (New England Biolabs) and 3000 Ci/mmol [γ-^32^P]ATP (PerkinElmer). The length and purity of the PCR product was confirmed by electrophoresis on a 1% agarose gel in TAE buffer (40 mM Tris-acetate pH 8.3/2 mM EDTA).

All DNase I protection reactions contained 10 fmol/µL ^32^P-5′-labeled 153 bp substrate in total volume of 20 µL of DNase reaction buffer (25 mM Tris-HCl pH 7.4/70 mM KCl/7 mM Mg Cl_2_/3 mM CaCl_2_/1 mM EDTA/1 mM β-mercaptoethanol/50 µg/mL BSA/7% glycerol) and were incubated at 25°C for 30 min after the addition of the Fis or Vap proteins for DNA binding. Samples contained 20–800 fmol/µL protein as indicated by the molar ratio of protein to DNA given in the figure legend. For VapBC-1 reactions, VapBC-1 was reconstituted from VapB-1 and VapC-1 proteins at a 3∶1 molar ratio by incubation for 20 min at room temperature prior to addition to the samples. This VapB-1 to VapC-1 ratio was shown in a previous study to fully inhibit VapC-1 ribonuclease activity, suggesting that all VapC-1 molecules were sequestered in VapBC-1 complexes [10]. Therefore, the molar concentration of VapBC-1 complexes in the reconstituted system was estimated as the concentration of VapC-1, since the complexes could not be directly measured. Following the binding incubation, 0.02 U DNase I (New England Biolabs) was added, and the 25°C incubation continued for 2 min. Reactions were quenched with 75 µL of DNase I stop solution (90% ethanol/220 mM sodium acetate/70 ng/µL yeast tRNA), mixed, and placed immediately in a dry ice/ethanol bath for 30 min. The nucleic acid was precipitated by centrifugation at 13000× g for 30 min at 4°C, the pellets were washed with cold 70% ethanol, then dried. The pellets were resuspended in 95% formamide/0.01% bromophenol blue/0.01% xylene cyanol and resolved on a 5% denaturing 19∶1 polyacrylamide gel in TBE (89 mM Tris-borate/1 mM EDTA). DNase I fragments of the ^32^P-5′-labeled strand were detected using a GE Healthcare Storm 845 phosphorimaging system.

### Electrophoretic mobility shift assays

A set of ^32^P-5′-labeled, 50 bp substrates were prepared for analysis of protein interaction with the *vapBC-1* locus control region. Complementary single-stranded oligonucleotides containing *vapB-1* translation initiation region (TIR) sequence or an upstream (US) sequence, shown to lack DNase I protection sites for Fis and the VapBC-1 complex, were obtained from Eurofins MWG Operon (Huntsville, AL). The sense strand of each 50 bp sequence, shown in Table 3, was ^32^P labeled, as described above, and annealed at a 1∶1.2 molar ratio with the unlabeled, complementary oligonucleotide by heating at 90°C for 3 min and cooling slowly to 4°C.

**Table 3:** Gel shift substrates.^a^

50TIR	5′-AAT GAT TAG TAT ATA CTT ATT AAA TAC ATA GTA TAT ACG AGA GGG TAA AT-3′
TG50TIR	5′-AAT GAT TAG TAT ATA CTT ATT AAA TAC ATA GTA GAT ACG AGA GGG TAA AT-3′
GC50TIR	5′-AAT GAT TAC TAT ATA CTT ATT AAA TAC ATA GTA TAT ACG AGA GGG TAA AT-3′
2M50TIR	5′-AAT GAT TAC TAT ATA CTT ATT AAA TAC ATA GTA GAT ACG AGA GGG TAA AT-3′
50US	5′-AAC AAC GGT AAT TTG ATC TTC TTA CTT GCA TAC AGC AAT TGA AAT GAT TA-3′

a Sense strand sequence of the 50 bp substrate shown with substitutions underlined.

For DNA binding reactions, all samples contained 1 fmol/µL ^32^P-labeled 50 bp substrate in total volume of 10 µL of EMSA reaction buffer (20 mM HEPES pH 7.5/100 mM NaCl/0.1 mM EDTA/1 mM dithiothreitol/50 µg/mL BSA/5% glycerol). Proteins were added at the protein∶DNA ratios indicated in the figure legends and incubated at 25°C for 30 min. VapBC-1 was reconstituted in a 3∶1 VapB-1 to VapC-1 molar ratio as described above. For the VapB-1 titration, VapC-1 was incubated with substrate at 25°C for 20 min, VapB-1 was added, and incubation was continued for 20 min prior to gel loading. Immediately following addition of 2.5 µL 50% glycerol buffer (50% glycerol/10 mM Tris-HCl pH 7.4/0.01% bromophenol blue/0.01% xylene cyanol) samples were loaded onto an 18 cm×16 cm×1.5 mm 5% native 29∶1 polyacrylamide gel containing 1% glycerol and 0.5× TBE buffer (44 mM Tris-borate/0.5 mM EDTA) that had been pre-electrophoresed for 30 min in 0.5× TBE/1% glycerol at 4°C. DNA species were resolved by electrophoresis at 20 mA for 45–55 min at 4°C and detected by phosphorimaging as above.

## Results

### Fis is subject to growth-phase regulation in NTHi

**Figure 1:**
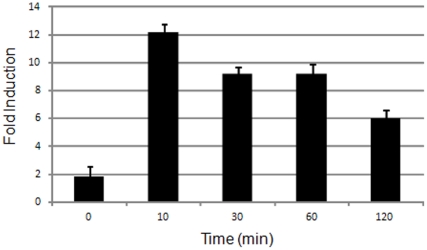
Expression of NTHi *fis* following nutrient upshift. Quantitative real-time PCR was performed for *fis* mRNA following nutrient upshift of NTHi wild type strain R2866. The zero time point is *fis* expression in the stationary phase culture prior to dilution into fresh media, while samples at 10, 30, 60, and 120 minutes indicate *fis* mRNA levels after nutrient upshift. Each time point included three biological replicates and three technical replicates. Error bars are standard deviations.

If Fis functions as an activator of *vapBC-1* expression, the cellular levels of *fis* transcript should correlate with growth phase increases in mRNA expression from the *vapBC-1* promoter. Although the regulation of Fis has been studied in other organisms, no data was available for the NTHi Fis. Therefore, quantitative real time-PCR was conducted to determine whether transcription of *fis* mRNA was induced upon nutrient upshift of NTHi. Using the *gyrA* gene as an endogenous control, relative expression of *fis* was examined at zero, 10, 30, 60, and 120 minutes following stationary phase culture dilution into fresh sBHI media (nutrient upshift) (Fig. 1). A dramatic increase in *fis* mRNA levels following nutrient upshift was observed. At ten minutes, *fis* expression was more than 10-fold that of the stationary phase culture, with a decrease at 120 minutes post-dilution, corresponding to the onset of exponential phase growth in NTHi. These results for NTHi *fis* expression were consistent with the pattern of growth phase regulation previously established for Fis in *E. coli* [11].

### Fis enhances gene expression from the *vapBC-1* locus control region upon nutrient upshift *in vivo*

**Figure 2:**
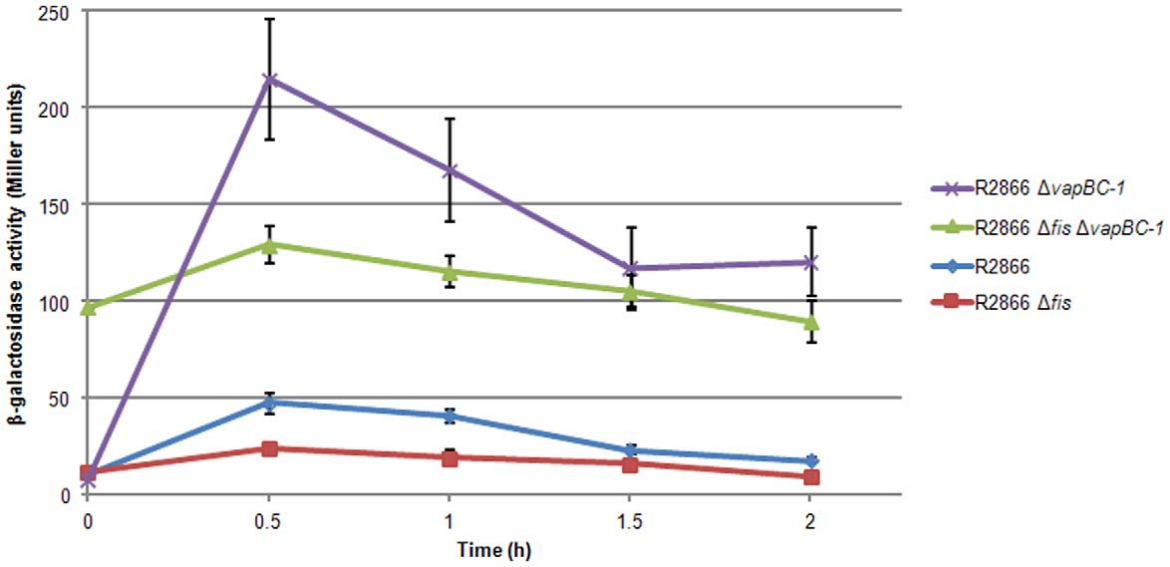
Expression from the NTHi *vapBC-1* promoter. β-galactosidase activity indicating *lacZ* expression under *vapBC-1* promoter control in wild type R2866 (diamonds), and R2866 mutants, Δ*fis* (squares), Δ*vapBC-1* (crosses), and Δ*fis* Δ*vapBC-1* (triangles), was measured every 30 minutes for 2 hours after nutrient upshift. The zero time point indicates expression in the stationary phase culture prior to dilution into fresh media. Each data point is the average of three independent assays performed in triplicate. Error bars are standard deviations.

To determine the effect of Fis and VapBC-1 proteins on *vapBC-1* expression, NTHi Δ*vapBC-1* and Δ*fis* mutants were constructed, along with a Δ*vapBC-1* Δ*fis* double mutant. The broad host-range plasmid pDD727, containing the *vapBC-1* promoter::*lacZ* fusion, was then conjugated into each of the wild type parent, Δ*vapBC-1*, Δ*fis*, and Δ*fis* Δ*vapBC-1* NTHi strains. Although designated the “*vapBC-1* promoter” for ease of reference, the pDD727 construct contains both the promoter of R2866 *vapBC-1* and the translation initiation region (TIR) of *vapB-1*, cloned as described above. Therefore, this protocol analyzes the genetic effects of Fis and Vap proteins on both the transcription and translation of the TA operon. Expression of *lacZ* from the *vapBC-1* promoter was monitored by β-galactosidase activity assays every 30 minutes for the first two hours following nutrient upshift (Fig. 2). The *vapBC-1* mutant expresses the activator Fis upon nutrient upshift, but not the VapBC-1 repressor complex, therefore transcription induction was the highest in this strain of the four strains analyzed. The wild type strain has both Fis and the VapBC-1 repressor complex, which in combination led to a regulated and lower expression of the *vapBC-1* operon. The Fis deletion strain has only the VapBC-1 repressor present, and resulted in the lowest transcription level. Finally, the Fis and VapBC-1 double deletion mutant has neither the activator (Fis) nor the repressor (VapBC-1), which led to unregulated constitutive transcriptional activity.

The presence of Fis in either the wild type or the Δ*vapBC-1* strain activated *lacZ* expression from the *vapBC-1* promoter upon nutrient upshift. The highest level of β-galactosidase activity was observed in the Δ*vapBC-1* mutant (which contained a functional *fis* gene), while activity was suppressed below wild-type levels in the Δ*fis* mutant (which contained a functional *vapBC-1* operon), supporting the hypothesis that Fis enhances *vapBC-1* expression during growth phase and the VapBC-1 protein complex autorepresses the *vapBC-1* locus control region. Interestingly, the Δ*fis* Δ*vapBC-1* double mutant showed little relative change in β-galactosidase activity upon nutrient upshift and maintained *lacZ* expression during stationary phase, suggesting that *vapBC-1* expression is constitutive in the absence of both VapBC-1 and Fis and that the locus is subjected to little or no growth phase-related transcriptional or translational regulation in the double mutant. These data also argue against other regulatory proteins interacting with the *vapBC-1* locus control region under the conditions of this assay.

### Fis and Vap proteins bind distinct sequences in the *vapBC-1* locus control region

**Figure 3:**
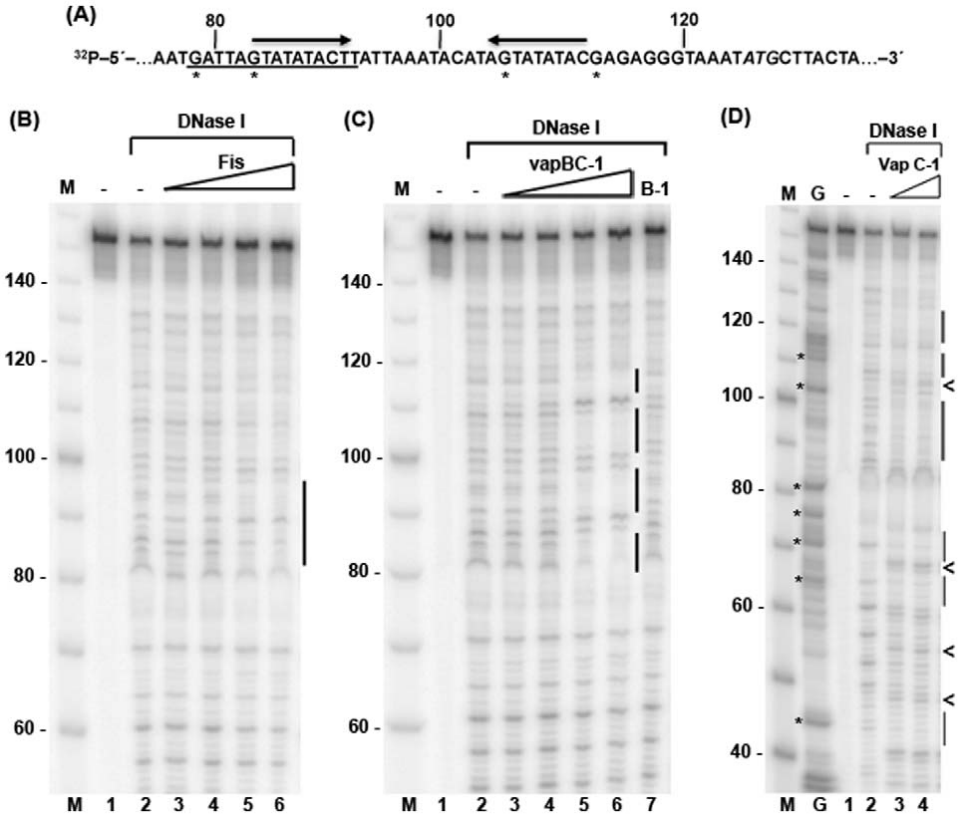
DNase I protection of the *vapBC-1* locus control region by Fis and Vap proteins. (A) The ^32^P-labeled sense strand of 153 bp DNA substrate containing *vapB-1* TIR and upstream sequence in the *vapBC-1* locus control region, is shown with numbers indicating the distance from the 5′-labeled end. The putative Fis site (*underline*), inverted repeat regions (*arrows*), *vapB-1* translation start ATG (*italics*), and G cleavage products (*) seen in (D, *lane G*) are noted. On each gel shown, a 10 bp DNA ladder (*lane M*), 153 bp substrate without protein (*lane 1*), and DNase I digest of the substrate (*lane 2*) are indicated. Gels show DNase I cleavage products from samples containing: (B) a Fis∶DNA molar ratio of 2∶1, 7.5∶1, 15∶1, or 30∶1 (*lanes 3–6*), (C) a VapBC-1∶DNA molar ratio of 2∶1, 7.5∶1, 15∶1, or 30∶1 (*lanes 3–6*) or 40∶1 VapB-1∶DNA (*lane 7*), and (D) VapC-1∶DNA molar ratio of 40∶1 or 80∶1 (*lanes 3* and *4*). Vertical bars indicate the DNase I footprint from protein binding. Arrows (<) in panel *D* indicate DNase I hypersensitive sites. The gels each represent one of two independent experiments.

The R2866 NTHi *vapB-1* TIR contains a putative Fis binding site that matches 13 of 15 residues of the *E. coli* Fis high-affinity consensus sequence [18], [19]. An inverted repeat sequence, which also may be utilized by regulatory proteins, overlaps a portion of the Fis site (Fig. 3A). The NTHi Fis protein shares 81% identity with the 97 amino acid *E. coli* Fis, but is divergent in the N-terminal region, where a proline insertion results in a 98 amino acid protein. Therefore, NTHi Fis is expected to display DNA binding properties similar to the *E. coli* protein and may utilize the putative site to mediate nutrient-dependent regulation of *vapBC-1*.

To analyze Fis and Vap protein interaction with the *vapBC-1* locus control region, we performed DNase I protection assays with a 153 bp substrate containing the *vapB-1* TIR and upstream locus control region sequence of R2866 *vapBC-1* used in the pDD727 construct for the *in vivo* assays. A single DNase I protection footprint for Fis was noted in the 80–100 base region on the 5′-labeled strand of the substrate (Fig. 3B). The footprint corresponds to the location of the putative Fis site in the TIR. These findings are consistent with a role for Fis in growth phase activation of *vapBC-1* expression.

The overexpression of wild-type VapC-1 is toxic to *E. coli*, and the toxin must be co-expressed with inhibitory VapB-1, which can co-purify in complexes with the toxin and limit the analysis of VapC-1 properties. To facilitate *in vitro* studies of VapC-1 in the absence of VapB-1, the ribonuclease function of the toxin was eliminated by an active site aspartate to asparagine substitution, forming D99N VapC-1. The purified D99N protein (henceforth referred to as VapC-1) retained its ability to associate with purified VapB-1, and VapBC-1 complexes were reconstituted by mixing of the antitoxin and toxin at a 3∶1 molar ratio. This preparation of VapC-1 allowed for the investigation of its role in the DNA binding activity of the TA pair.

DNase I protection footprints for VapBC-1 were observed in nucleotide regions 82–87, 90–98, 102–109, and 112–117 in the substrate, all of which lie within 50 bases of the ATG translation start site for *vapB-1*, which is at position 125 from the labeled end of the substrate (Fig. 3C). The footprints correspond to the inverted repeat region of the substrate, and the 82–98 site overlaps with the single Fis footprint, indicating that VapBC-1 and Fis may compete for binding of the *vapB-1* TIR in the regulation of *vapBC-1* expression. VapB-1 alone showed no DNA interaction when present at high concentration, suggesting that it must be bound to VapC-1 in order to interact with DNA (*lane 7*, Fig. 3C). Interestingly, VapC-1 alone exhibited several sites of DNase I protection on the substrate, with footprints overlapping those of VapBC-1 but also appearing at unique sites within the 40–70 nucleotide region, upstream of the TIR (Fig. 3D). Unlike VapBC-1, VapC-1 binding also induced DNase I hypersensitive sites, suggesting that several VapC-1 molecules may interact with each substrate molecule, leaving short stretches of DNA exposed to the nuclease. Higher VapC-1 concentrations were required than in the VapBC-1 samples to observe DNase I protection, likely because VapC-1 occupied more sites than the TA complex. A Maxam-Gilbert chemical digestion of the 153 bp substrate was performed to obtain a banding pattern for strong cleavage at G and weak cleavage at A [25] (*lane G*, Fig. 3D). The digest aided the location of the VapC-1 within the substrate and revealed that the substrate DNase I fragments migrated slightly faster in the gel than the corresponding marker fragments (comparing *lanes M* and *G* where asterisks indicate substrate positions 48, 68, 79, 84, 106, and 114). These results indicate that VapC-1 possesses DNA binding activity, and the toxin may be responsible for VapBC-1 interaction with the TIR of *vapB-1*.

### Fis, VapC-1 and VapBC-1 interact with the translation initiation region of *vapB-1*

**Figure 4:**
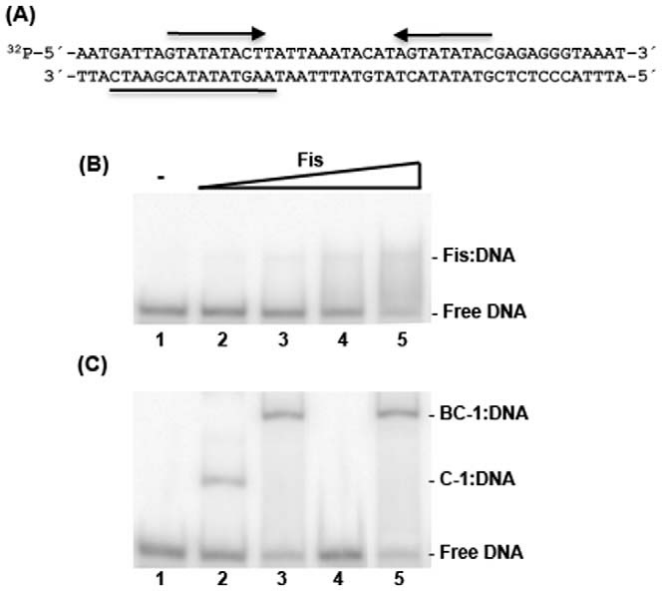
Fis, VapC-1 and VapBC-1 bind the *vapB-1* TIR. (A) The sequence of the 50TIR indicating the putative Fis binding site (*underline*) and the inverted repeat regions (*arrows*). (B) Gel shift products from samples containing 50TIR alone (*lane 1*) and 10, 30, 150 or 600 molar ratios of Fis to DNA. (C) Products from samples containing 50TIR alone (*lane 1*), VapC-1 (*lane 2*), VapC-1 followed by VapB-1 (*lane 3*), VapB-1 (*lane 4*), or the reconstituted VapBC-1 complex (*lane 5*). VapB-1 and VapC-1 are present in a 150∶1 molar ratio to 50TIR. In VapBC-1 samples, VapB-1 and VapC-1 are at a 3∶1 molar ratio, with a VapC-1∶DNA molar ratio of 150∶1. This ratio is reported since VapC-1 is the DNA binding protein and the actual amount of VapBC-1 complexes cannot be determined. The identity of each band is noted at the right of each gel. Each gel represents one of two independent experiments.

Since Fis, VapBC-1 and VapC-1 share footprints in the *vapB-1* TIR of the *vapBC-1* locus control region, 50 base pair DNA substrates containing the TIR (50TIR), the TIR with a T to G mutation in the inverted repeat (TG50TIR), the TIR with a G to C mutation at the −7 position in the putative Fis site (GC50TIR), or sequence upstream of the TIR that lacked Fis and VapBC-1 footprints (50US) were studied by gel shift analysis for Fis and Vap protein binding (Table 2). Initial experiments were performed to monitor the binding of Fis, VapBC-1 or individual Vap proteins with the 50TIR substrate (Fig. 4A). Fis decreased the mobility of 50TIR in a concentration-dependent manner; however, the Fis-bound DNA did not migrate as a distinct band, suggesting that the Fis∶DNA complexes were labile under the gel conditions (Fig. 4B). The VapC-1 toxin and VapBC-1 complex bound stably to 50TIR, and VapC-1∶DNA complexes were shifted to a DNA mobility corresponding to that of VapBC-1∶DNA complexes when VapB-1 was added following VapC-1 pre-incubation with the substrate (Fig. 4C). The VapB-1 antitoxin displayed no DNA binding activity, likewise no DNA interaction was observed with a purified VapB-1 protein lacking the N-terminal polyhistidine tag, confirming that the His tag was not interfering with VapB-1 association with DNA (*data not shown*). These observations were consistent with the DNase I protection seen for each protein, but the Fis findings indicate that the polyhistidine tag on the purified Fis compromised DNA binding or that Fis may not interact specifically with the *vapB-1* TIR.

### NTHi Fis exhibits non-specific binding with the *vapBC-1* locus control region

**Figure 5:**
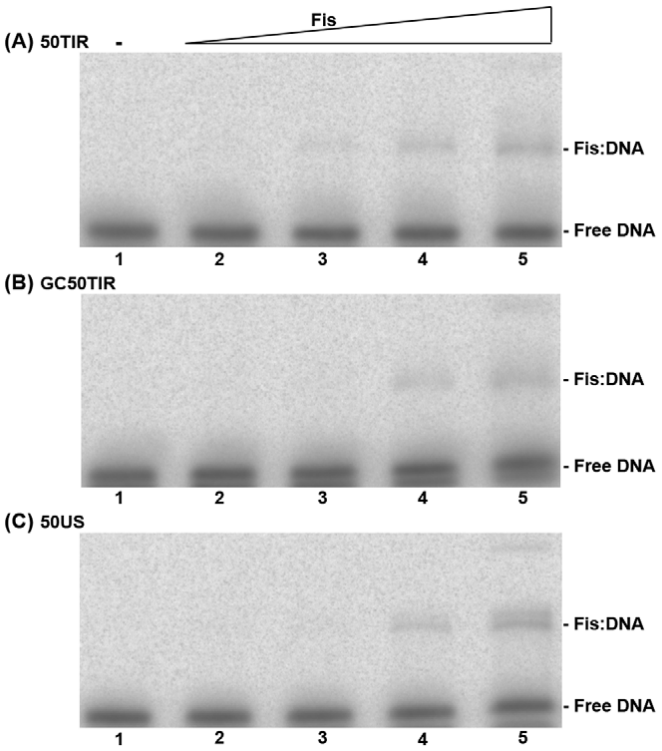
Fis interacts non-specifically with the *vapB-1* TIR. Gel shift products from a titration of Fis, lacking a polyhistidine tag, with (A) 50TIR, (B) GC50TIR or (C) 50US are shown. The gels show the products of the following samples: DNA only (*lane 1*) and the addition of Fis at 10, 30, 90 or 300 molar ratios with the DNA substrate (*lanes 2–5*). The identity of each band is noted at the right of each gel. Each gel represents one of two independent experiments.

The presence of a single DNase I protection site for Fis suggested that Fis binding to the *vapB-1* TIR was specific; however, the results above indicate a low affinity of Fis for this sequence. To more carefully examine Fis interaction with the *vapBC-1* locus control region, purified Fis lacking the N-terminal polyhistidine tag was prepared to study Fis binding to 50TIR, as well as the GC50TIR and 50US substrates, to which Fis binding was expected to be diminished or absent. While a polyhistidine tag did not alter VapB-1 binding properties, the N-terminal His tag on Fis appeared to be partly responsible for the instability of Fis complexes with 50TIR observed in Fig. 4, as a distinct Fis∶DNA band was resolved for Fis without the tag (Fig. 5). Surprisingly, Fis displayed similar affinity to all three substrates. These results indicate that, while Fis obviously upregulates *vapBC-1* expression upon nutrient upshift *in vivo*, the protein may do so indirectly through its effects on DNA structure and not by high-affinity binding to specific sequences in the locus control region.

### VapBC-1 specifically interacts with the *vapB-1* translation initiation region

**Figure 6:**
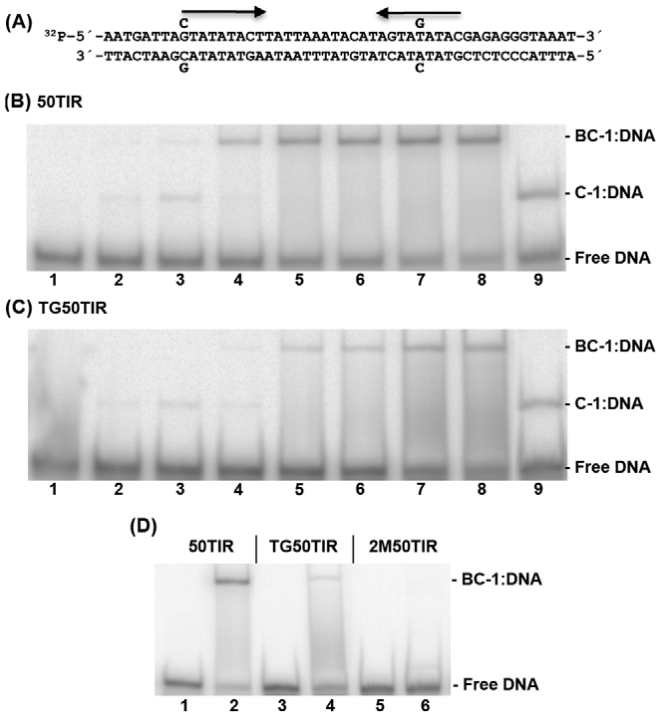
VapBC-1 specifically interacts with the *vapB-1* TIR. (A) The sequence of the 50TIR is shown with *arrows* indicating the inverted repeat regions and bases above and below the sequence indicating the position of the G to C and T to G substitutions in the TG50TIR and 2M50TIR substrates (see Table 3). Gel shift products from a titration of VapBC-1 with (B) 50TIR or (C) TG50TIR are shown. The lanes in panels *B* and *C* contain the following samples: DNA only (*lane 1*), the addition of VapBC-1 at a VapC-1∶DNA ratio of 10, 25, 50, 100, 200, 400 or 800 molar ratio with the DNA substrate (*lanes 2–8*), and VapC-1 only at an 800∶1 protein to DNA ratio (*lane 9*). (D) A comparison of VapBC-1 binding at a 400∶1 molar ratio with each DNA substrate (*lanes 2, 4*, and *6*). *Lanes 1, 3*, and *5* contain only DNA. The identity of each band is noted at the right of each gel. Each gel represents one of three independent experiments.

The VapBC-1 complex protected four areas of the *vapB-1* TIR from DNase I digestion. The footprints were within a 40-base region that contained both the putative Fis site and an inverted repeat. As the inverted repeat may serve as a recognition sequence for the complex, a T to G transversion was introduced in one half of the repeat, generating TG50TIR (Fig. 6A). The substitution diminished binding of VapBC-1 by an average of 5 to 10-fold over a concentration range of VapBC-1, implicating the inverted repeat in VapBC-1 recognition and possible autoregulation of the *vapBC-1* operon (Fig. 6B). Interestingly, a band corresponding to VapC-1∶DNA complexes was observed for both 50TIR and TG50TIR at the low concentrations of VapBC-1. The band shifted to the BC-1∶DNA mobility as the concentration of protein increased. This observation suggests that VapBC-1 formation is not required prior to DNA binding, and that VapB-1 can associate with VapC-1∶DNA complexes. A double-mutant TIR substrate, 2M50TIR, was created by including a G to C transversion in the TG50TIR substrate, which altered the upstream half of the inverted repeat. Nucleotide changes in both sides of the inverted repeat completely abolished VapBC-1 interaction with the TIR sequence (Fig. 6D). VapC-1 binding to the 2M50TIR substrate was reduced only by half compared to that observed with 50TIR (*data not shown*). This result shows that optimal binding of VapBC-1 to the *vapB-1* TIR depends on the inverted repeat. VapBC-1 utilization of both sides of the repeat suggests that the complex may bind as a dimer, and the requirement for VapB-1 in conferring complex specificity indicates that both VapB-1 and VapC-1 contribute to the DNA binding surface of the complex.

**Figure 7:**
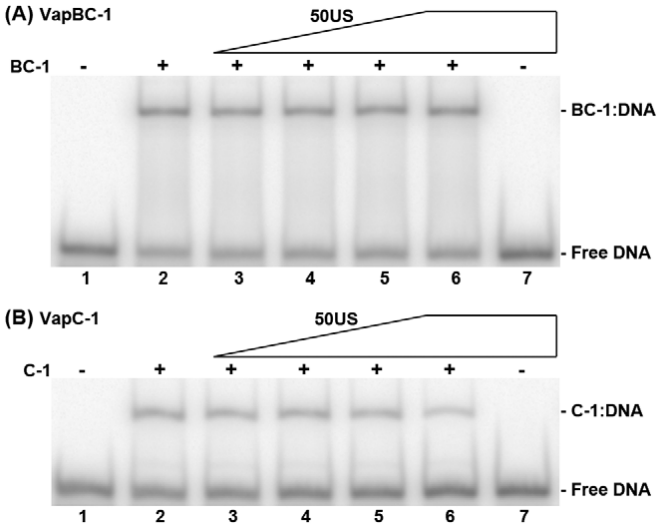
Competition binding of 50TIR and 50US by Vap proteins. Gel shift products from addition of 50US substrate into samples containing 50TIR substrate and either (A) VapBC-1 or (B) VapC-1 at a 150∶1 molar ratio of VapC-1 protein to DNA. VapB-1 and VapC-1 are at a 3∶1 molar ratio in the VapBC-1 samples, but VapC-1∶DNA molar ratios are reported since VapC-1 is the DNA binding protein and the actual amount of VapBC-1 complexes cannot be determined. The gels show the products of the following samples: 50TIR without protein (*lane 1*), protein with only 50TIR (*lane 2*), the addition of a 1∶1, 5∶1, 10∶1 or 50∶1 molar ratio of cold 50US:50TIR (*lanes 3–6*), and DNA only at 50∶1 molar ratio of 50US:50TIR (*lane 6*). The identity of each band is noted at the right of each gel. Each gel represents one of two independent experiments.

To assess whether VapBC-1 binding was specific to the *vapB-1* TIR, the 50US sequence, which contained no detectable VapBC-1 DNase I footprints, was analyzed for VapBC-1 and VapC-1 binding competition with the 50TIR substrate. A titration of unlabeled 50US substrate into samples with constant amounts of VapBC-1 and 50TIR failed to diminish the BC-1∶DNA band (Fig. 7A). Indeed, no interaction of VapBC-1 with a labeled 50US was observed up to a 300 to 1 molar ratio of protein to DNA (data not shown). On the other hand, 50US did disrupt binding of VapC-1 to the 50TIR substrate, indicating that VapC-1 alone does not have high affinity for the *vapB-1* TIR (Fig. 7B). These results confirmed that VapBC-1 interacts specifically with the *vapB-1* TIR and that VapB-1 may target the complex to the inverted repeat as a recognition site in suppressing *vapBC-1* expression.

### VapB-1 targets VapC-1 to the *vapB-1* translation initiation region

**Figure 8:**
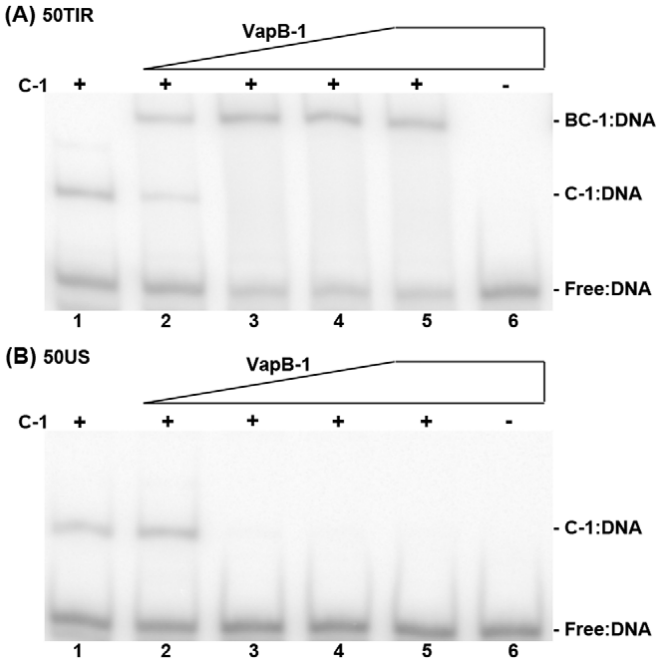
VapB-1 targets VapC-1 to the *vapB-1* translation initiation region. Gel shift products from a titration of VapB-1 into samples following VapC-1 binding to (A) 50TIR or (B) 50US at a 100∶1 molar ratio of protein to DNA. The gels show the products of the following samples: VapC-1 and DNA without VapB-1 (*lane 1*), the addition of Vap B-1 at a 50, 100, 200 or 300 molar ratio with the DNA substrate (*lanes 2–5*), and VapB-1 only at a 300∶1 protein to DNA ratio (*lane 6*). The identity of each band is noted at the right of each gel. Each gel represents one of two independent experiments.

Both the DNase I results and initial experiments with 50TIR indicate that VapC-1 alone binds the locus control region of *vapBC-1*; however, the role of VapC-1 binding relative to that of the VapBC-1 complex is unclear, especially since the toxin is thought to act primarily as a ribonuclease that is inhibited by VapB-1. Since VapB-1 has no DNA binding property of its own, this raises the possibility that VapC-1 facilitates DNA binding by the VapBC-1 complex, and that VapB-1 binds to VapC-1 for DNA interaction. The observations above indicate that VapBC-1 formed in the cytosol may specifically bind the *vapB-1* TIR or that VapB-1 may bind to a VapC-1:TIR complex to enhance DNA affinity. To elucidate whether VapB-1 interacts with VapC-1 on DNA and how this interaction may affect VapBC-1 specificity, VapB-1 was added to samples following VapC-1 preincubation with either the 50TIR or 50US substrates (Fig. 8). VapC-1 alone forms stable complexes with both substrates, revealing that VapC-1 lacks specificity for the *vapB-1* TIR. As observed in the previous experiments, VapB-1 shifts the 50TIR substrate from the C-1∶DNA mobility to a slower BC-1∶DNA complex band (Fig. 8A), but interestingly, it causes the dissociation of VapC-1 from 50US (Fig. 8B). VapB-1 alone did not associate with either substrate, even at its highest concentration. These findings indicate that VapB-1 alters the affinity of VapC-1 for DNA and confers specificity for the *vapB-1* TIR to the VapBC-1 complex.

## Discussion

Bacteria often encounter microenvironments in the host that include suboptimal conditions, such as nutrient limitation, oxidative or antibiotic stress. Organisms that cannot respond to these challenges in a constructive fashion may not survive until conditions improve. One straightforward way for a nonsporulating species to endure poor environments is to enter a reversible bacteriostatic state. Since most drugs target essential biosynthetic pathways that are not active during periods of dormancy, growth arrest confers many benefits upon a bacterium, including very low metabolic energy requirements as well as nonspecific antibiotic tolerance. The key to utilizing such a state effectively, however, lies in the ability of the organism to transition back to replication and growth when stress is relieved. This novel mechanism of growth modulation has been attributed to the action of TA loci, and the *vapBC* locus has been shown to regulate growth arrest by inhibiting translation in a number of bacteria [26]–[28]. Although *vapBC* loci from various organisms share little nucleotide homology, many show conservation of function in their ribonuclease activity [7], [29], [30].

The current work focused on understanding the regulation of the *vapBC-1* TA locus in NTHi, a bacterium responsible for a number of chronic mucosal infections in humans. Given the growth phase regulation of its operon and its role in gene regulation in *E. coli*, NTHi *fis* expression and its effect on expression the *vapBC-1* locus were studied. We have shown that the NTHi *fis* gene, like its homolog in *E. coli*, is dramatically upregulated by nutrient upshift, suggesting a role for NTHi Fis in growth phase dependent gene regulation [11]. Through β-galactosidase activity studies of *vapBC-1* promoter activity in Δ*vapBC-1*, Δ*fis*, and Δ*fis* Δ*vapBC-1* NTHi strains, we have found that *fis* is necessary for nutrient upshift activation of the *vapBC-1* promoter driving *lacZ* and that the genomic *vapBC-1* attenuates promoter activity. In fact, the highest level of *vapBC-1* promoter activity was observed in the Δ*vapBC-1* strain. While a DNA sequence homologous to the *E. coli* Fis consensus site was identified in the translation initiation region (TIR) of *vapB-1*, Fis binding to this sequence was not specific, indicating that Fis upregulates *vapBC-1* expression indirectly by altering DNA structure [13]. The autoregulatory effects of VapBC-1 implied by the β-galactosidase assay appear to be mediated by recognition of the *vapBC-1* operon by the TA pair. We demonstrated that the VapBC-1 complex binds specifically to the *vapB-1* TIR of the *vapBC-1* locus control region by utilizing an inverted repeat sequence. Interestingly, we discovered that the VapC-1 toxin possesses DNA binding activity, which appears to facilitate interaction of the VapBC-1 complex with the *vapBC-1* locus. VapC-1 can associate independently with DNA, while VapB-1 does not bind DNA under any of our experimental conditions. Some antitoxins do not bind individually to their cognate promoters in the absence of their toxins. For example, no DNA binding activity could be detected for two RelB-like antitoxin proteins in *M. tuberculosis* [31]. We have previously shown that VapC-1 is a ribonuclease [10], and its ability to independently bind double-stranded nucleic acid suggests that it may act upon RNA secondary structure. The finding that VapC-1 binds DNA is remarkable considering that many TA loci are autoregulated by the antitoxin product, with the toxin acting as a co-repressor [32]. Finally, the VapB-1 antitoxin conferred binding specificity to VapC-1, as it caused VapC-1 to dissociate from sites outside of the *vapB-1* TIR but formed a stable complex with VapC-1 on the *vapB-1* TIR. Taken together, these results suggest novel roles for the *vapBC-1* TA pair in regulation of growth arrest with Fis serving to indirectly enhance *vapBC-1* expression after growth stimulation.

**Figure 9:**
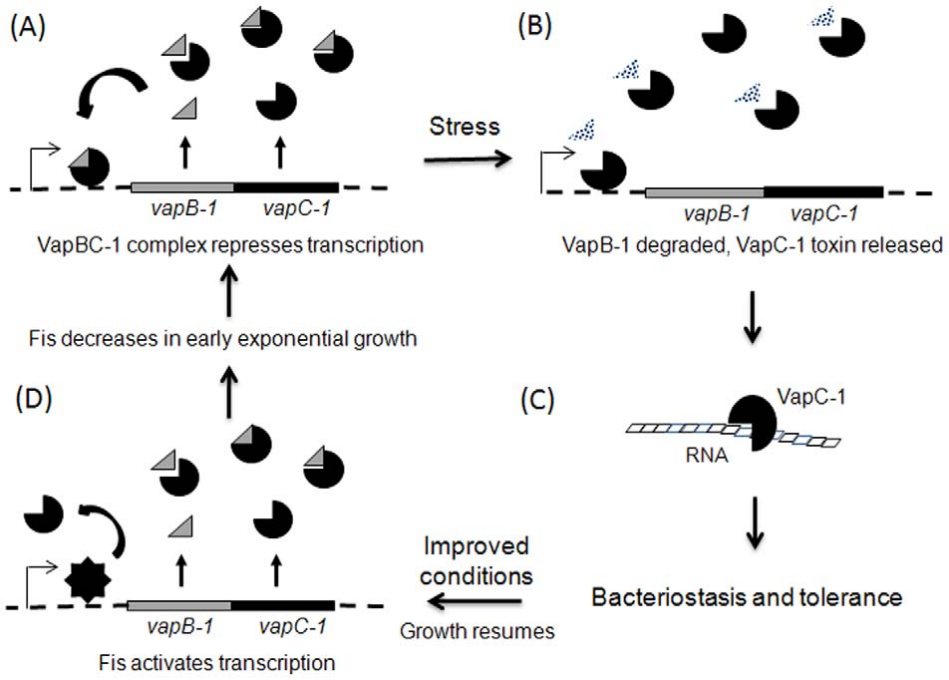
Model for the regulation of the *vapBC-1* locus. (A) During colonization under favourable conditions, the VapBC-1 complex binds to and autorepresses TA operon transcription. (B) Stress induces Lon and Clp proteases that degrade VapB-1, releasing active VapC-1 toxin. (C) The ribonuclease activity of VapC-1 facilitates a state of bacteriostasis, resulting in nonspecific antibiotic tolerance. (D) Upon improved conditions, Fis activates *vapBC-1* operon transcription, displacing any bound VapC-1. Fis levels decrease in early exponential growth, allowing the VapBC-1 complex to bind and restore transcriptional equilibrium.

Previously, the role of VapB-1 in NTHi survival was thought to be the binding and inactivation of VapC-1 ribonuclease activity. However, our findings suggest a new paradigm for TA locus autoregulation and for antitoxin activity in bacterial survival of nutrient stress (Fig. 9). In NTHi, Fis appears to stimulate *vapBC-1* expression on resumption of growth, as is observed for other bacterial species. We have confirmed that VapBC-1 suppresses its own operon when *fis* expression drops in early log phase growth (Fig. 9A). However, VapBC-1 autoregulation of the TA operon is facilitated through the DNA binding property of the VapC-1 toxin, with the antitoxin serving to target the TA complex to the *vapB-1* TIR. VapC-1 may associate with VapB-1 in the cytosol prior to DNA interaction or bind to DNA alone, suggesting that DNA binding by the toxin may also play a role in attenuating its ribonuclease activity. When nutrient deprivation or environmental stress arrests NTHi growth, VapB-1 would be degraded by Lon and Clp proteases, freeing VapC-1 from VapBC-1 complexes and releasing the inhibition on the VapC-1 ribonuclease (Fig. 9B) [33]. The toxin could remain on the *vapB-1* TIR or dissociate to degrade the cellular RNA pool to inhibit translation (Fig. 9C). Since the VapC-1 interaction with *vapB-1* TIR appears less stable in the absence of VapB-1, it may be readily displaced by Fis-induced structural changes in the promoter when conditions favor the resumption of NTHi growth (Fig. 9D). This model is consistent with another study that demonstrated reversible growth inhibition in the absence of the VapB antitoxin when the transcription of the VapC toxin was first induced, then subsequently blocked, in a *M. smegmatis* strain containing a deletion in its single *vapBC* homologue [28]. Indeed, if this model serves to explain *vapBC-1* regulation in NTHi, drugs that stabilize the interaction of VapB-1 and VapC-1 or inhibit the ribonuclease function of VapC-1 should provide a new and effective approach for the treatment of chronic NTHi infections.

## References

[1] Murphy TF, Apicella MA (1987). Nontypeable *Haemophilus influenzae*: a review of clinical aspects, surface antigens, and the human immune response to infection.. Rev Infect Dis.

[2] Murphy TF, Brauer AL, Schiffmacher AT, Sethi S (2004). Persistent colonization by *Haemophilus influenzae* in chronic obstructive pulmonary disease.. Am J Respir Crit Care Med.

[3] Lewis K (2007). Persister cells, dormancy and infectious disease.. Nat Rev Microbiol.

[4] Pandey DP, Gerdes K (2005). Toxin-antitoxin loci are highly abundant in free-living but lost from host-associated prokaryotes.. Nucl Acid Res.

[5] Ramage HR, Connolly LE, Cox JS (2009). Comprehensive functional analysis of *Mycobacterium tuberculosis* toxin-antitoxin systems: implications for pathogenesis, stress responses, and evolution.. PLoS Genet.

[6] Fatica A, Tollervey D, Dlakic M (2004). PIN domain of Nob1p is required for D-site cleavage in 20S pre-rRNA.. RNA.

[7] Arcus VL, McKenzie JL, Robson J, Cook GM (2011). The PIN-domain ribonucleases and the prokaryotic VapBC toxin-antitoxin array.. Protein Eng, Design & Select.

[8] Zhu L, Sharp JD, Kobayashi H, Woychik NA, Inouye M (2010). Noncognate *Mycobacterium tuberculosis* toxin-antitoxins can physically and functionally interact.. J Biol Chem.

[9] Marchler-Bauer A, Anderson JB, Chitsaz F, Derbyshire MK, DeWeese-Scott C (2009). CDD: specific functional annotation with the Conserved Domain Database.. Nucl Acids Res.

[10] Daines DA, Wu MH, Yuan SY (2007). VapC-1 of nontypeable *Haemophilus influenzae* is a ribonuclease.. J Bacteriol.

[11] Ball CA, Osuna R, Ferguson KC, Johnson RC (1992). Dramatic changes in Fis levels upon nutrient upshift in *Escherichia coli*.. J Bacteriol.

[12] Schneider R, Lurz R, Luder G, Tolksdorf C, Traver A (2001). An architectural role of the *Escherichia coli* chromatin protein FIS in organising DNA.. Nucl Acids Res.

[13] Opel ML, Aeling KA, Holmes WM, Johnson RC, Benham CJ (2004). Activation of transcription initiation from a stable RNA promoter by a Fis protein-mediated DNA structural transmission mechanism.. Mol Microbiol.

[14] Hirsch M, Elliott T (2005). Fis regulates transcriptional induction of RpoS in *Salmonella enterica*.. J Bacteriol.

[15] Grainger DC, Hurd D, Goldberg MD, Busby SJ (2006). Association of nucleoid proteins with coding and non-coding segments of the Escherichia coli genome.. Nucleic Acids Res.

[16] Skoko D, Yoo D, Bai H, Schnurr B, Yan J (2006). Mechanism of chromosome compaction and looping by the Escherichia coli nucleoid protein Fis.. J Mol Biol.

[17] Hengen PN, Bartram SL, Stewart LE, Schneider TD (1997). Information analysis of Fis binding sites.. Nucl Acids Res.

[18] Shao Y, Feldman-Cohen LS, Osuna R (2008). Functional characterization of the *Escherichia coli* Fis-DNA binding sequence.. J Mol Biol.

[19] Feldman-Cohen LS, Shao Y, Meinhold D, Miller C, Colon W (2006). Common and variable contributions of Fis residues to high-affinity binding at different DNA sequences.. J Bacteriol.

[20] Kostrewa D, Granzin J, Stock D, Choe H-W, Labahn J (1992). Crystal structure of the factor for inversion stimulation FIS at 2.0 Å resolution.. J Mol Biol.

[21] Mason KM, Munson RS, Bakaletz LO (2003). Nontypeable *Haemophilus influenzae* gene expression induced in vivo in a chinchilla model of otitis media.. Infect Immun.

[22] Casadaban MJ, Chou J, Cohen SN (1980). In vitro gene fusions that join an enzymatically active beta-galactosidase segment to amino-terminal fragments of exogenous proteins: *Escherichia coli* plasmid vectors for the detection and cloning of translational initiation signals.. J Bacteriol.

[23] Daines DA, Smith AL (2001). Design and construction of a *Haemophilus influenzae* conjugal expression system.. Gene.

[24] Miller JH (1972). Experiments in molecular genetics..

[25] Maxam AM, Gilbert W (1977). A new method for sequencing DNA.. Proc Natl Acad Sci U S A.

[26] Winther KS, Gerdes K (2009). Ectopic production of VapCs from *Enterobacteria* inhibits translation and *trans*-activates YoeB mRNA interferase.. Mol Microbiol.

[27] Buts L, Lah J, Dao-Thi M-H, Wyns L, Loris M (2005). Toxin-antitoxin modules as bacterial metabolic stress managers.. TIBS.

[28] Robson J, McKenzie JL, Cursons R, Cook GM, Arcus VL (2009). The *vapBC* operon from *Mycobacterium smegmatis* is an autoregulated toxin-antitoxin module that controls growth via inhibition of translation.. J Mol Biol.

[29] Ahidjo BA, Kuhnert D, McKenzie JL, Machowski EE, Gordhan BG (2011). VapC toxins from Mycobacterium tuberculosis are ribonucleases that differentially inhibit growth and are neutralized by cognate VapB antitoxins.. PLOS One.

[30] Miallau L, Faller M, Chiang J, Arbing M, Guo F (2009). Structure and proposed activity of a member of the VapBC family of toxin-antitoxin systems: VapBC-5 from *Mycobacterium tuberculosis*.. J Biol Chem.

[31] Yang M, Gao C, Wang Y, Zhang H, He ZG (2010). Characterization of the interaction and cross-regulation of three Mycobacterium tuberculosis RelBE modules.. PLOS One.

[32] Bailey SES, Hayes F (2009). Influence of operator site geometry on transcriptional control by the YefM-YoeB toxin-antitoxin complex.. J Bacteriol.

[33] Gronlund H, Gerdes K (1999). Toxin-antitoxin systems homologous with relBE of Escherichia coli plasmid P307 are ubiquitous in prokaryotes.. J Mol Biol.

